# Ultrasound-assisted extraction of polyphenols from *Lonicera japonica* leaves and their α-glucosidase inhibition

**DOI:** 10.3389/fnut.2025.1542217

**Published:** 2025-01-29

**Authors:** Gang Li, Chunming Bao, Hao Zhang, Lu Bai

**Affiliations:** ^1^School of Food Science, Henan Institute of Science and Technology, Xinxiang, China; ^2^Henan Institute of Metrology, Zhengzhou, China

**Keywords:** honeysuckle leaves, polyphenol extracts, α-glucosidase, enzyme inhibition, fluorescence

## Abstract

**Introduction:**

The search for natural bioactive compounds that act as α-glucosidase inhibitors is a central focus in diabetes treatment research.

**Methods:**

This study utilized ultrasonic-assisted extraction to optimize the extraction of polyphenols from honeysuckle leaves through response surface methodology (RSM). Enzyme inhibition and fluorescence quenching experiments were conducted to examine the inhibitory activity and binding behavior of the extracted polyphenols.

**Results:**

The results indicated that the optimal conditions for polyphenol extraction were as follows: ethanol concentration, 64%, liquid–solid ratio, 45 mL/g, ultrasonic power, 700 W, ultrasonic time, 6 min. Under these conditions, the yield of polyphenols from honeysuckle leaves was 9.16 ± 0.19%, which closely aligns with the predicted value of 9.18%. The total phenolic content of the polyphenol extracts was 20.6 ± 0.67%, with chlorogenic acid and luteoloside contents measuring 5.65 ± 0.40% and 2.51 ± 0.14%, respectively. The inhibitory effect of polyphenol extracts (IC_50_, 0.14 ± 0.01 mg/mL) on α-glucosidase was better than that of chlorogenic acid (IC_50_, 0.55 ± 0.02 mg/mL). Fluorescence quenching experiments indicated that the polyphenol extracts interact with α-glucosidase, resulting in alterations to the microenvironment of amino acid residues.

**Discussion:**

This interaction can reduce the binding affinity between the substrate and α-glucosidase, thereby contributing to the objective of lowering postprandial hyperglycemia. Our research results can provide reference for the development and utilization of honeysuckle leaves.

## 1 Introduction

Currently, diabetes, as one of the predominant chronic diseases, extensively and persistently affects people’s lives (1–6). Controlling postprandial hyperglycemia is a critical approach in diabetes treatment, which is achieved by inhibiting the activity of carbohydrate-related hydrolases (α-amylase and α-glucosidase). Inhibitors of these enzymes slow glucose absorption in the intestine, thereby mitigating the postprandial increase in blood glucose levels. Classic antidiabetic drugs such as miglitol, acarbose, and voglibose function as α-glucosidase inhibitors (7). Their molecular structures resemble those of disaccharides or oligosaccharides, enabling them to bind to the carbohydrate-binding site of α-glucosidase. This binding reduces the interaction between α-glucosidase and carbohydrates, resulting in delayed digestion and absorption of carbohydrates, ultimately lowering postprandial hyperglycemia. However, substantial evidence indicates that the use of these medications can lead to various adverse effects, including abdominal pain, diarrhea, and hepatotoxicity (8, 9). In recent years, people have begun to focus on the development and utilization of natural products, which are low-cost and relatively safe. Džamić and Matejić (10) summarized the natural active compounds used in diabetes treatment, including plant extracts, carotenoids, polyphenols, saponins, flavonoids, phytosterols, and other bioactive compounds, which represent a rich source for the development of therapeutic and preventive drugs for diabetes.

Honeysuckle (*Lonicera japonica* Thunb.) is an important medicinal and edible plant. Its flowers can be utilized in the production of traditional Chinese medicine formulations and serves as an industrial raw material for the extraction of chlorogenic acid. Despite the annual increase in its market price, the branches and leaves of honeysuckle are largely discarded as non-medicinal components. Research indicates that the leaves and flowers of honeysuckle exhibit significant homogeneity in their primary bioactive constituents. In particular, the leaves exhibit considerable activity, even surpassing that of the floral components, and can serve as an alternative or supplementary source to flowers (11). The leaves are rich in polyphenolic compounds, including chlorogenic acid and luteoloside (12), and their and pharmacological effects and active components are comparable to those of the flower buds. Chlorogenic acid, the primary active component in the leaves of honeysuckle, possesses significant potential for development and application. Agunloye et al. (13) found that chlorogenic acid can reduce the activity of enzymes associated with the pathogenesis of hypertension, demonstrating favorable antihypertensive and cardioprotective properties. Additionally, chlorogenic acid has shown promising efficacy in reducing blood glucose levels, suggesting its potential as a preventive agent for type 2 diabetes (14). Luteoloside exerts neuroprotective effects through mechanisms that reduce the apoptosis rate of nerve cells, as well as through antioxidant and anti-inflammatory actions (15). China possesses abundant and diverse resources of honeysuckle. Effective utilization and development of the polyphenols from honeysuckle leaves could yield broad application prospects.

Ultrasound-assisted extraction technology, recognized for its efficiency, rapidity, and environmental benefits, plays an increasingly significant role in the extraction of bioactive compounds from plants (16). This method utilizes mechanical vibrations generated by ultrasound to facilitate the extraction of active components from plant samples. The cavitation effect induced by these vibrations, combined with elevated temperature and pressure, enhances extraction efficiency, reduces extraction time, and minimizes the use of harmful solvents (17). Traditional water extraction methods for polyphenols exhibit limitations such as low extraction efficiency and challenges in separation. Ultrasound-assisted extraction improves the yield of polyphenols while simultaneously reducing extraction time and minimizing solvent consumption. Xu et al. (18) demonstrated that, compared to conventional maceration and Soxhlet extraction methods, ultrasound-assisted extraction significantly increases the yield of antioxidants and decreases extraction time.

In view of this, the present study employed response surface methodology (RSM) to optimize the process conditions for ultrasound-assisted extraction of polyphenols, and enzyme inhibition and fluorescence quenching experiments were conducted to investigate the enzyme inhibitory activity and interactions of polyphenols from honeysuckle leaves. The findings of this study can serve as a reference for the development and utilization of honeysuckle leaves.

## 2 Materials and methods

### 2.1 Materials and chemicals

The leaves of honeysuckle (variety, Baijin 2) were harvested from the experimental field in Xinxiang. The powder of honeysuckle leaves after drying and crushing was stored in the laboratory of the School of Food Science at Henan Institute of Science and Technology (Xinxiang, China). α-glucosidase (from yeast) and p-nitrophenyl-α-D-glucopyranoside (PNPG) were purchased from Aladdin (Shanghai, China). All other chemicals were of analytical grade.

### 2.2 Ultrasonic-assisted extraction of polyphenols from honeysuckle leaves

Honeysuckle leaves were dried at a constant temperature of 50°C until no significant weight change was observed. The dried leaves were subsequently crushed and sieved to obtain honeysuckle leaf powder, which was stored in a dark environment at refrigerated temperatures. One gram of powdered honeysuckle leaves was weighed and placed in a 50 mL conical flask, followed by the addition of a specific volume of an ethanol-water mixed solution to ensure thorough mixing. The mixture underwent ultrasonic treatment in an ultrasonic cleaner, followed by centrifugation at 6,000 rpm for 10 min, after which the supernatant was collected.

### 2.3 Determination of total phenolic content

According to the methods described in reference (19), with slight modifications. First, 300 μL of the aforementioned collected supernatant was added to a colorimetric tube, followed by the addition of distilled water to reach a total volume of 10 mL. Subsequently, 0.5 mL of Folin–Ciocalteu reagent was added, and the mixture was shaken for 5 min. Immediately thereafter, 5 mL of 5% Na_2_CO_3_ solution was added, and distilled water was used to adjust the final volume to 25 mL. The mixture was thoroughly mixed and allowed to stand for 90 min. Absorbance was measured at a wavelength of 750 nm, and the total phenolic content of the supernatant was calculated using a chlorogenic acid standard curve. The yield of polyphenols was calculated by Equation 1.


(1) $$Y=\frac{C_{1}}{C_{0}}\times 100\%$$

where, *Y* was the polyphenol yield of honeysuckle leaves, %; *C*_1_ was the total amount of polyphenols in the supernatant, mg; *C*_0_ was the mass of the honeysuckle leaf raw material, mg.

### 2.4 Single factor experiment of ultrasonic-assisted extraction

The experiment evaluated the yield of polyphenols from honeysuckle leaves, examining the effects of ethanol concentration (10, 30, 50, 70, 90%), liquid–solid ratio (10, 20, 30, 40, 50 mL/g), ultrasonic time (2, 4, 6, 8, 10, 12 min), and ultrasonic power (280, 420, 560, 700 W) on the polyphenol yield.

### 2.5 Optimization experiment of ultrasonic-assisted extraction based on RSM

Based on the results of the single factor experiments, RSM was employed to further optimize the conditions for ultrasonic-assisted extraction. Four factors were selected as independent variables: ethanol concentration (A), liquid–solid ratio (B), ultrasonic time (C), and ultrasonic power (D), with the response variable being the yield of polyphenols (Y).

### 2.6 Determination of total phenolic content in extracts

The supernatant obtained from the aforementioned process was subjected to rotary evaporation to remove ethanol, followed by freeze-drying to obtain the polyphenol extract. 2 mg/mL methanolic solution of the extract was prepared and the total phenolic content was determined according to the method described in section 2.3.

### 2.7 Determination of chlorogenic acid and luteoloside content

According to the reference method and slightly modified (20). Determination of chlorogenic acid and luteoloside using a Waters Alliance HPLC system (e2695 separating module) (Waters Co., MA, United States). The extract solution of 2 mg/mL was prepared with methanol, and the column was Waters C18 (4.6 mm × 250 mm, 5 μm). Acetonitrile was mobile phase A, 0.04% glacial acetic acid solution was mobile phase B, the volume flow rate was 1.0 mL/min, the column temperature was 30°C, the injection volume was set at 20 μL, and the gradient elution was performed for 0–10 min, A: 3–15%, B: 97–85%. 10–31 min, A: 15–30%, B: 85–70%, detection wavelength was 348 nm.

### 2.8 Determination of α-glucosidase inhibitory effect

According to the method described in the reference (21), the inhibitory activity of α-glucosidase by sample solutions at different concentrations was determined, with chlorogenic acid as the positive control and PNPG as the substrate. The reaction was carried out in PBS buffer solution (0.1 mol/L, pH 6.8). Respectively, 1 mL of chlorogenic acid solution and 1 mL of polyphenol extract solution were uniformly mixed with 1 mL of α-glucosidase solution (0.2 U/mL). The mixed solutions were incubated in a water bath at 37°C for 10 min. Subsequently, added PNPG (1 mL, 1 mmol/L) and incubate the resulting mixed solution in a water bath at 37°C for 20 min. Finally, 1 mL ethanol was quickly added to terminate the reaction, and the final volume was 4 mL. The absorbance value was measured at 405 nm (*A*_1_). The control group (*A*_0_) was treated with 1 mL of PBS buffer solution instead. The inhibitory activity (*R*) of α-glucosidase was calculated according to Equation 2:


(2) $$R=\frac{A_{0}-A_{1}}{A_{0}}\times 100\%$$

### 2.9 Determination of binding behavior

According to Geng et al. (21), the interaction of polyphenol extracts with α-glucosidase was determined using a fluorescence quenching method. PBS buffer solution (50 mmol/L, pH 6.8) was used to prepare 2 U/mL α-glucosidase solution and different concentrations of polyphenol extract solutions (0, 10, 20, 30, 40, 50 mg/mL). Four milliliters of the aforementioned enzyme solution was uniformly mixed with 1 mL of the polyphenol extract solution and stabilized at 30°C for 10 min. The fluorescence spectra were scanned using an Agilent Cary Eclipse Fluorescence Spectrophotometer (Santa Clara, CA). The scanning conditions were set as follows: excitation wavelength/280 nm, scanning range/300–450 nm, slit width/5 nm, and scanning voltage/750 kV.

### 2.10 Determination of synchronous and 3D fluorescence spectra

Referring to the method of He et al. (22) with slight modifications, the conformational changes of α-glucosidase were identified by measuring the synchronous and 3D spectral behaviors of tyrosine and tryptophan residues. The reaction system of enzyme-polyphenol extract was constructed according to the method described in section 2.9. The synchronous fluorescence spectra were recorded at Δ*λ* = 15 nm/Ex = 265–350 nm and Δ*λ* = 60 nm/Ex = 220–350 nm. The 3D fluorescence spectra of the sample solution were recorded with excitation wavelengths ranging from 220 to 300 nm and emission wavelengths ranging from 300 to 450 nm. The scanning rate was set at 9,600 nm/min.

### 2.11 Statistical analysis

Each experiment was conducted with three repetitions, and the resulting data underwent error analysis. Graphs were generated using Origin 8.0 software. Response surface data analysis was conducted using Design-Expert software.

## 3 Results and discussion

### 3.1 Analysis of results from single factor experiments

Ultrasound-assisted extraction technology is an efficient, rapid, and environmentally friendly method that increasingly plays a significant role in extracting bioactive compounds from plants. In examining the conditions of the ultrasonic-assisted extraction process, numerous parameters influence the yield of polyphenols, including the type of extraction solvent, the liquid–solid ratio, ultrasonic power, and ultrasonic time. Given the potential applications of honeysuckle leaf polyphenols in the food industry, ethanol, a food-grade solvent, was selected as the extraction solvent. Furthermore, the liquid–solid ratio is directly related to cost-effectiveness, making it an important evaluation metric. The intensity of cavitation effects, determined by ultrasonic power and time, facilitates the release of polyphenols from plant tissues into the solvent, thereby influencing extraction efficiency. Consequently, this study examined the impact of these four factors on the extraction yield of polyphenols. The initial conditions for the fixed factors were established as follows: ethanol concentration 30%, liquid–solid ratio 30 mL/g, ultrasonic time 6 min, and ultrasonic power 560 W.

#### 3.1.1 The impact of ethanol concentration

**Figure 1:**
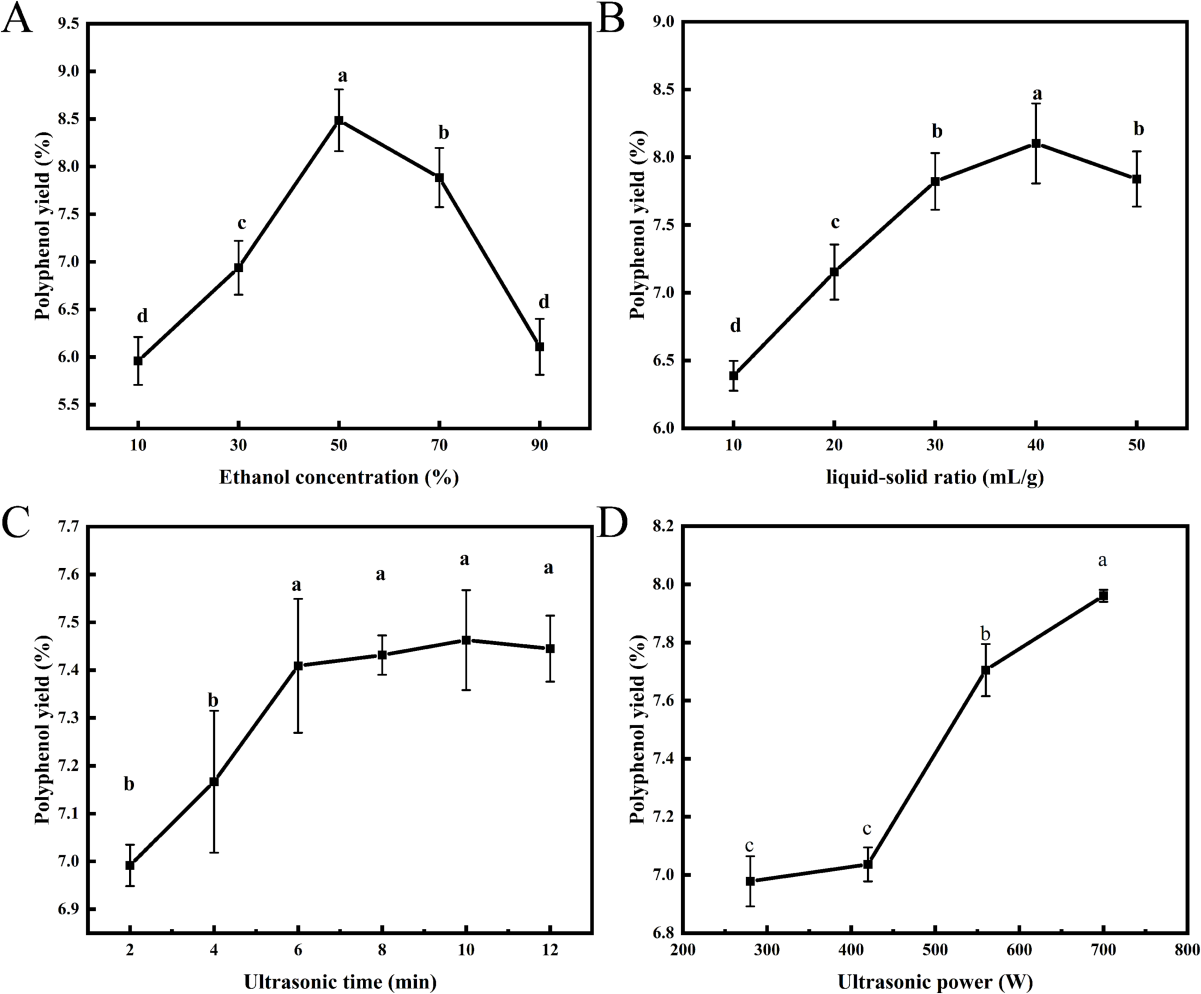
Effects of ethanol concentration **(A)**, liquid–solid ratio **(B)**, ultrasonic time **(C)** and ultrasonic power **(D)** on the yield of polyphenols.

Water enhances the swelling of plant materials, while ethanol disrupts the binding between solutes and the plant matrix (23). Figure 1A illustrated the effect of ethanol concentration on polyphenol yield, demonstrating that as ethanol concentration increases, polyphenol yield initially rises before subsequently declining. The highest yield of polyphenols from honeysuckle leaves, at 8.49%, was achieved at an ethanol concentration of 50%. Zhou et al. (24) reported a similar phenomenon when extracting polyphenols from the seed coat of the red sword bean. This trend may be attributed to insufficient ethanol concentration, which hinders penetration through plant cells. Conversely, excessively high concentrations increase the polarity of the mixture, thereby reducing interactions with polyphenolic compounds and resulting in lower yields of the target components. According to the principle of “like dissolves like,” the solubility of the target compound varies with changes in solvent polarity (25). Consequently, ethanol concentrations ranging from 30 to 70% were selected for subsequent RSM studies.

#### 3.1.2 The impact of liquid–solid ratio

The efficiency of polyphenol extraction is significantly influenced by the liquid–solid ratio. An inadequate amount of extraction solvent can result in incomplete extraction, while an excessive amount leads to wastage. Consequently, determining an appropriate liquid–solid ratio is essential. Figure 1B illustrated the effect of the liquid–solid ratio on polyphenol yield. As the liquid–solid ratio increased, the yield of polyphenols initially rose, followed by a subsequent decline. At a liquid–solid ratio of 40 mL/g, the maximum yield of polyphenols reached 8.10%. This outcome may be attributed to the increased liquid–solid ratio, which can dilute the concentration of target components in the solution, thereby facilitating the hydration and swelling of honeysuckle leaf powder and promoting the diffusion of constituents within the plant tissue. However, an excessively high liquid–solid ratio may complicate subsequent processing steps, thereby reducing overall efficiency. Similar experimental results have been reported in the extraction of polyphenols from pomegranate flowers (26). Therefore, a liquid–solid ratio of 30–50 mL/g was selected for subsequent RSM studies.

#### 3.1.3 The impact of ultrasonic time

When the ethanol concentration and liquid–solid ratio are held constant, the yield of polyphenols is closely related to ultrasonic time and power. Ultrasonic vibration facilitates the rapid penetration of the solvent into the extract, thereby maximizing the dissolution of polyphenols within the solvent. Figure 1C illustrated the effect of ultrasonic time on the yield of polyphenols. Within the range of 2 to 6 min, the yield of polyphenols significantly increased with the prolongation of ultrasonic time (*p* < 0.05). After 6 min, the yield of polyphenols tended to level off, with no significant changes (*p* > 0.05). Prolonging ultrasonic time facilitated the release of active ingredients; however, as time progressed, the osmotic pressure inside and outside the leaves of honeysuckle reached equilibrium, leading to a gradual stabilization of the dissolution rate (27). Although the yield of polyphenols slightly increases with further extension of ultrasonic time, this is unnecessary. Therefore, to reduce energy consumption, ultrasonic time of 4–8 min was selected for optimization studies in subsequent RSM studies.

#### 3.1.4 The impact of ultrasonic power

Ultrasonic waves generate high-frequency vibrations that can create tiny bubbles in solvents, which burst instantaneously, resulting in localized conditions of high temperature and pressure. This phenomenon disrupts cell walls, cell membranes, and other plant cell structures, thereby promoting the release of target compounds from plant tissues into the solvent and enhancing extraction efficiency (28). Figure 1D illustrated the effect of varying ultrasonic power on the yield of polyphenols from honeysuckle leaves. As ultrasonic power increased, the yield of polyphenols also rose, reaching a maximum of 7.96% at 700 W. The progressively enhanced cavitation effect intensifies the energy surrounding the bubbles, making ultrasonic-assisted extraction more effective in disrupting cellular structures compared to conventional methods, thus facilitating the release of polyphenols from honeysuckle leaf powder into the solvent. Therefore, an ultrasonic power range of 420–700 W was selected for subsequent RSM studies.

### 3.2 RSM optimization of ultrasonic-assisted extraction process

Based on the results of single-factor experiments, an RSM experiment was designed with the yield of polyphenolic extracts Y (%) as the response variable. The experiment consisted of 27 experimental points, including three central points, to optimize the extraction process. The RSM experimental design and results were presented in Table 1.

**Table 1:** RSM experimental design and results.

RSM experiment	A (%)	B (mL/g)	C (min)	D (W)	Y (%)
1	70 (1)	40 (0)	6 (0)	420 (−1)	7.58 ± 0.14
2	30 (−1)	40 (0)	6 (0)	700 (1)	7.63 ± 0.11
3	50 (0)	40 (0)	4 (−1)	700 (1)	8.58 ± 0.14
4	50 (0)	40 (0)	4 (−1)	420 (−1)	7.57 ± 0.16
5	50 (0)	40 (0)	8 (1)	700 (1)	9.17 ± 0.10
6	50 (0)	40 (0)	6 (0)	560 (0)	8.79 ± 0.15
7	70 (1)	40 (0)	4 (−1)	560 (0)	8.36 ± 0.09
8	30 (−1)	30 (−1)	6 (0)	560 (0)	7.17 ± 0.16
9	30 (−1)	40 (0)	6 (0)	420 (−1)	6.75 ± 0.07
10	30 (−1)	40 (0)	8 (1)	560 (0)	7.68 ± 0.21
11	50 (0)	40 (0)	8 (1)	420 (−1)	7.69 ± 0.20
12	70 (1)	50 (1)	6 (0)	560 (0)	8.61 ± 0.20
13	50 (0)	50 (1)	6 (0)	420 (−1)	7.28 ± 0.09
14	50 (0)	30 (−1)	6 (0)	420 (−1)	7.17 ± 0.08
15	30 (−1)	50 (1)	6 (0)	560 (0)	7.44 ± 0.11
16	50 (0)	40 (0)	6 (0)	560 (0)	8.94 ± 0.12
17	70 (1)	30 (−1)	6 (0)	560 (0)	7.65 ± 0.15
18	70 (1)	40 (0)	6 (0)	700 (1)	9.06 ± 0.13
19	70 (1)	40 (0)	8 (1)	560 (0)	8.86 ± 0.14
20	50 (0)	30 (−1)	4 (−1)	560 (0)	7.63 ± 0.09
21	50 (0)	50 (1)	4 (−1)	560 (0)	8.17 ± 0.18
22	50 (0)	50 (1)	6 (0)	700 (1)	8.61 ± 0.21
23	50 (0)	30 (−1)	8 (1)	560 (0)	7.88 ± 0.13
24	30 (−1)	40 (0)	4 (−1)	560 (0)	7.83 ± 0.09
25	50 (0)	40 (0)	6 (0)	560 (0)	8.86 ± 0.16
26	50 (0)	30 (−1)	6 (0)	700 (1)	7.89 ± 0.14
27	50 (0)	50 (1)	8 (1)	560 (0)	8.33 ± 0.24

**Figure 2:**
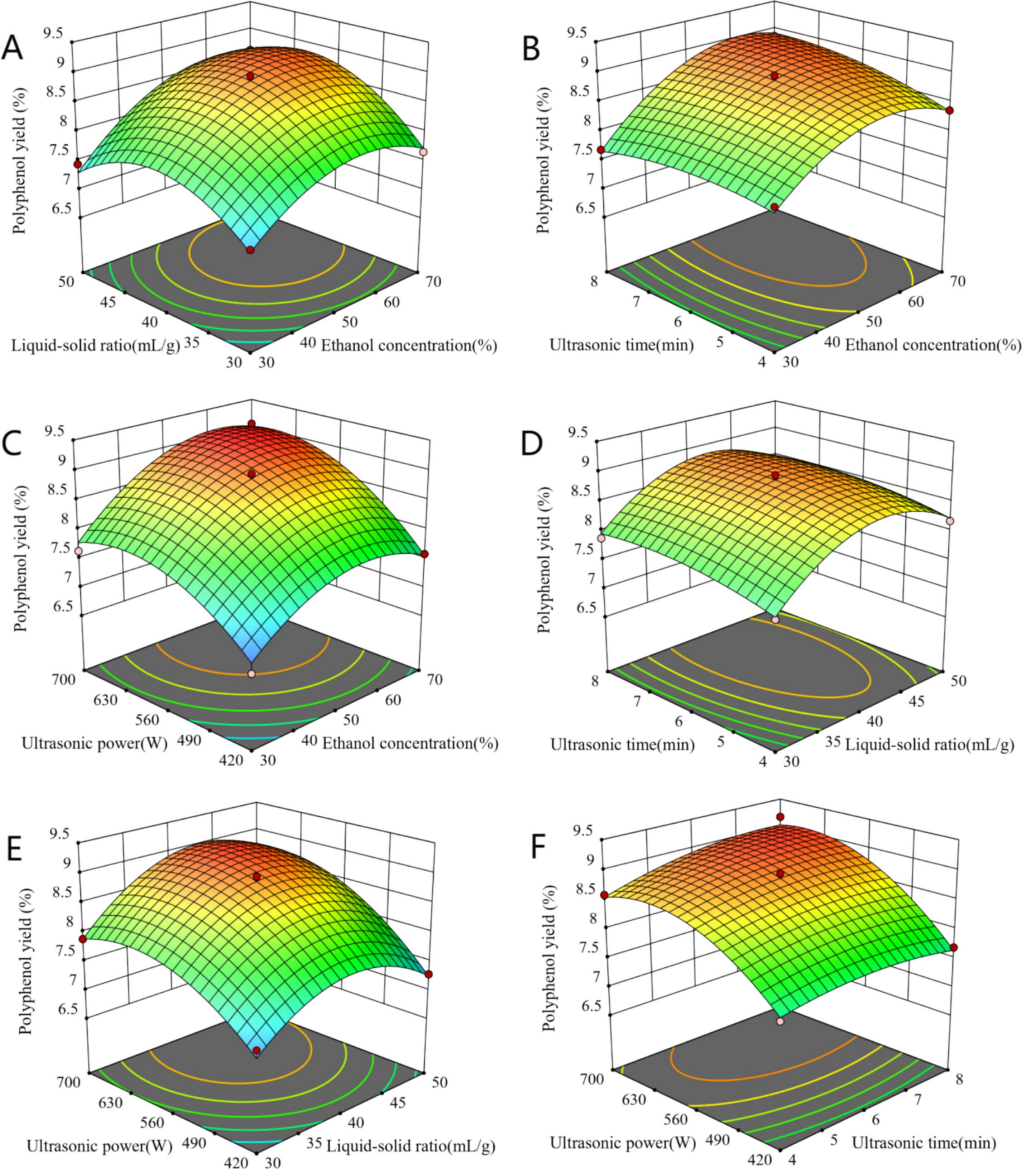
The interaction of ethanol concentration and liquid-solid ratio **(A)**, ethanol concentration and ultrasonic time **(B)**, ethanol concentration and ultrasonic power **(C)**, liquid-solid ratio and ultrasonic time **(D)**, liquid-solid ratio and ultrasonic power **(E)**, ultrasonic time and ultrasonic power **(F)** on polyphenol yield.

The results of the ANOVA are presented in Table 2. The model demonstrated high significance (*p* < 0.0001), while the lack of fit term was not significant (*p* = 0.2959). The mean square value of the fitted model equation yielded an *R*^2^ of 0.9845 and an adjusted *R*^2^ of 0.9664. These data indicated that the model fit was adequate to make reasonable predictions within the scope of the experiment. With the yield (Y) as the response variable, the regression equation obtained was: Y = 8.86 + 0.4679A + 0.2553B + 0.1233C + 0.5753D + 0.1699AB + 0.1611 AC + 0.1486 AD − 0.0244 BC + 0.1535BD + 0.1178CD − 0.5455A^2^ − 0.6450B^2^ − 0.1538C^2^ − 0.4994D^2^. Linear terms (A, B, C, D), interactive terms (AB, AC, AD, BD), and quadratic terms (A^2^, B^2^, C^2^, D^2^) significantly influenced polyphenol yield (*p* < 0.05). As shown in Figure 2, to predict the association between the dependent and independent variables, we utilized a regression equation to generate a 3D response surface plot. By comparing *F*-values and the curvatures of the 3D response surfaces, we determined that the interaction between AB was the most significant, followed by AC.

**Table 2:** Results of analysis of variance.

Source	Sum of squares	df	Mean square	*F*-value	*p*-value
Model	11.38	14	0.8132	54.40	<0.0001
A	2.63	1	2.63	175.74	<0.0001
B	0.7823	1	0.7823	52.33	<0.0001
C	0.1824	1	0.1824	12.20	0.0044
D	3.97	1	3.97	265.74	<0.0001
AB	0.1154	1	0.1154	7.72	0.0167
AC	0.1038	1	0.1038	6.94	0.0218
AD	0.0883	1	0.0883	5.91	0.0317
BC	0.0024	1	0.0024	0.1587	0.6974
BD	0.0942	1	0.0942	6.31	0.0274
CD	0.0555	1	0.0555	3.71	0.0781
A^2^	1.59	1	1.59	106.18	<0.0001
B^2^	2.22	1	2.22	148.41	<0.0001
C^2^	0.1261	1	0.1261	8.44	0.0132
D^2^	1.33	1	1.33	88.97	<0.0001
Residual	0.1794	12	0.0149		
Lack of fit	0.1672	10	0.0167	2.75	0.2959
Pure error	0.0122	2	0.0061		
Cor total	11.56	26			
Std. dev.	0.1223	C.V. %	1.52	Adj *R*^2^	0.9664
Mean	8.04	*R* ^2^	0.9845	Pred *R*^2^	0.9143

Based on the experimental results, the optimal extraction conditions were predicted using a second-order polynomial equation: ethanol concentration of 63.5%, liquid–solid ratio of 45.4 mL/g, ultrasonic time of 5.69 min, and ultrasonic power of 670.9 W. To facilitate practical applications, validation experiments were conducted under the following conditions: ethanol concentration of 64%, liquid–solid ratio of 45 mL/g, ultrasonic time of 6 min, and ultrasonic power of 700 W. The repeated experiments yielded a polyphenol extraction rate of 9.16 ± 0.19%, which closely approximated the predicted value of 9.18%, thereby confirming the reliability of the model.

### 3.3 Analysis of total phenol, chlorogenic acid and luteoloside content

**Figure 3:**
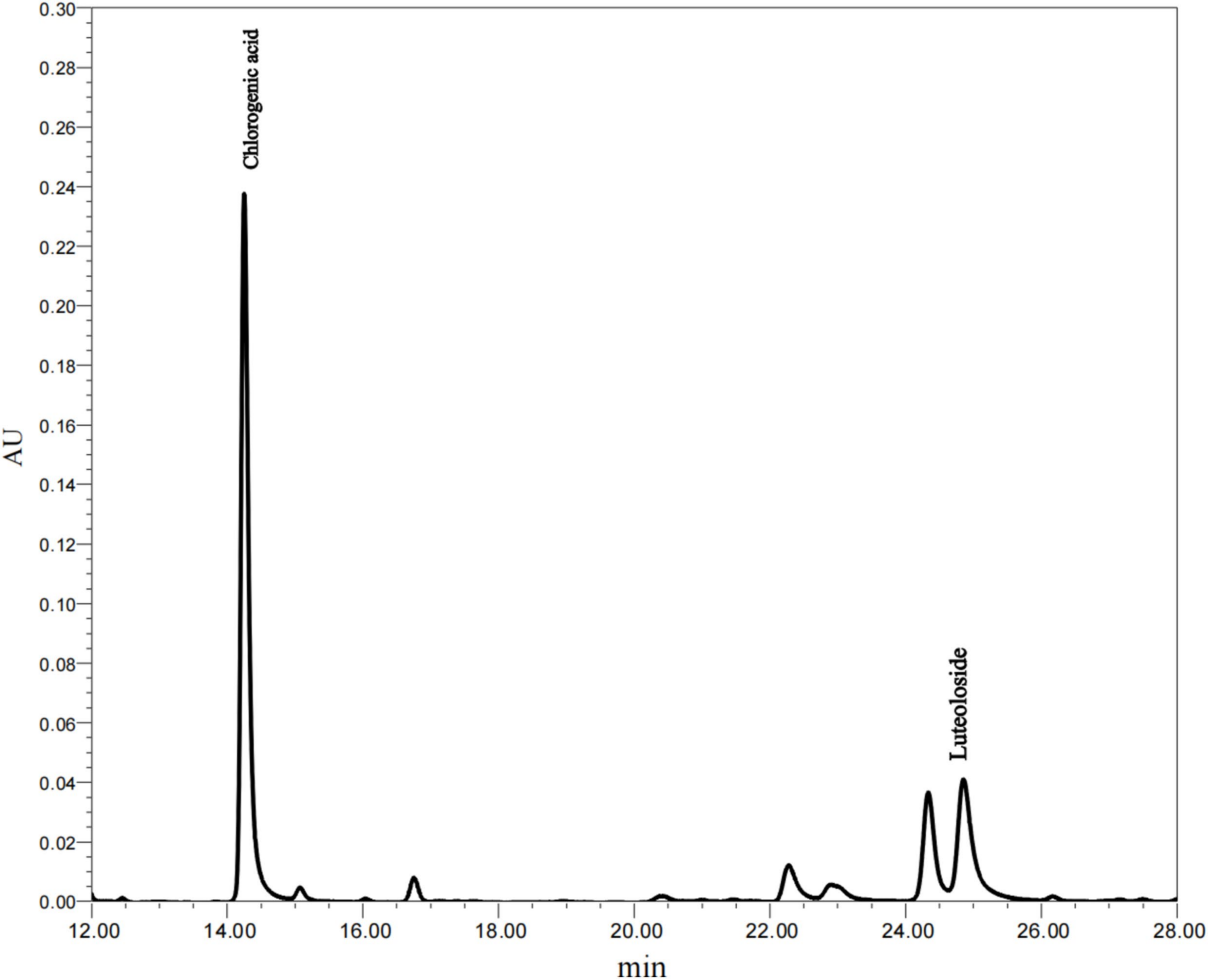
HPLC profile of the polyphenol extract.

Honeysuckle is abundant in bioactive compounds, primarily phenolic acids and flavonoids. Among these, chlorogenic acid is the most prominent phenolic acid, while luteoloside is the principal flavonoid (29, 30). According to the Chinese Pharmacopoeia, these two polyphenolic compounds are not only the primary active constituents of honeysuckle but also serve as quality control markers for this herb. As shown in Figure 3, the primary polyphenolic compounds identified in the leaf extracts were chlorogenic acid and luteoloside. As indicated in Table 3, the total phenolic content of honeysuckle leaves was measured at 20.6 ± 0.67%. The chlorogenic acid content in the extract was found to be 5.65 ± 0.40%, while the luteoloside content was 2.51 ± 0.14%. Yue et al. (29) reported that the leaves and buds of honeysuckle are rich in medicinal components, which can be utilized as raw materials for the extraction of chlorogenic acid and luteoloside, thus preventing waste.

**Table 3:** The polyphenol content of extract.

Polyphenol extract	Polyphenol content (%)
Total phenols	20.6 ± 0.67^a^
Chlorogenic acid	5.65 ± 0.40^b^
Luteoloside	2.51 ± 0.14^c^

Different letters in the same column represent significant differences between data (*p* < 0.05).

### 3.4 The inhibitory effect on α-glucosidase

**Figure 4:**
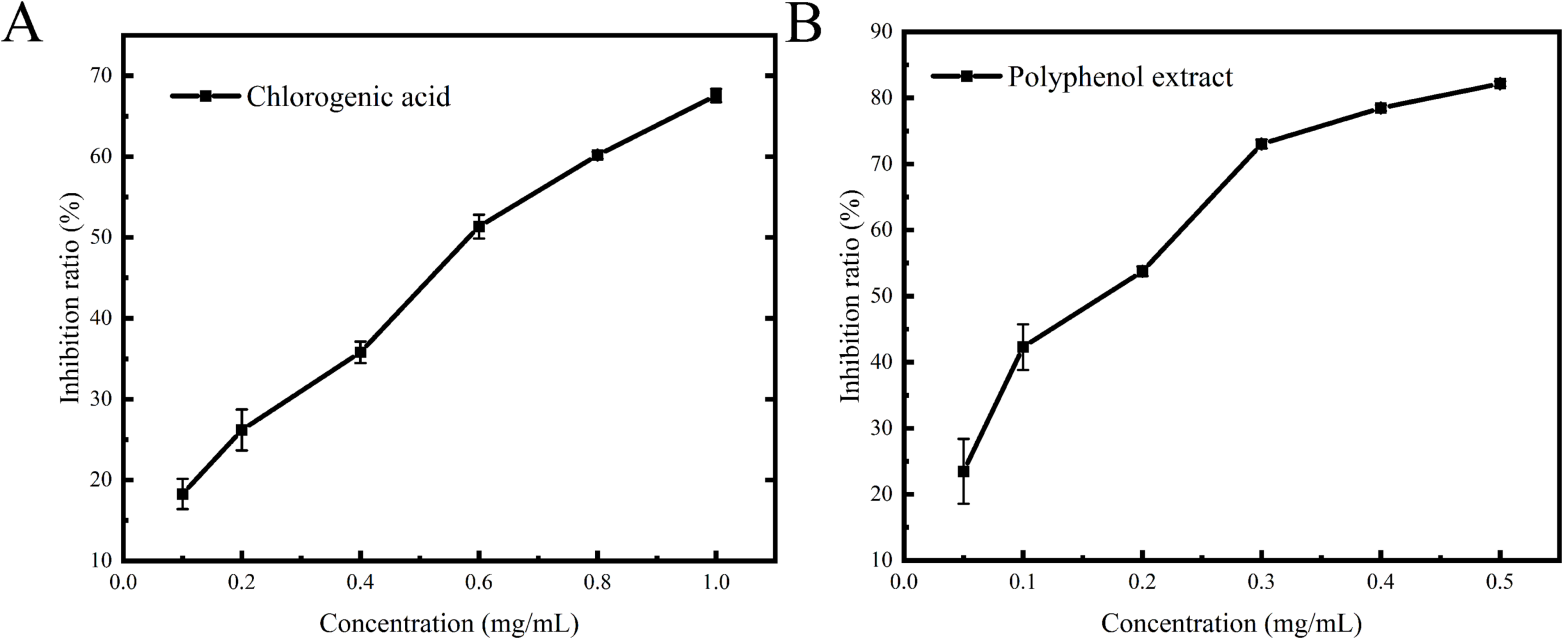
Effects of chlorogenic acid **(A)** and polyphenol extract **(B)** on α-glucosidase inhibition.

The analysis of the polyphenolic composition revealed that the extracts from the leaves of honeysuckle predominantly comprised chlorogenic acid and luteoloside, along with other polyphenolic compounds. Previous pharmacological studies have demonstrated that chlorogenic acid possesses significant antioxidant, anti-inflammatory, and antibacterial properties without any cytotoxicity (31–33), while luteoloside exhibits notable neuroprotective effects (34). These polyphenolic compounds from honeysuckle leaves (chlorogenic acid and luteoloside) exhibit favorable biological properties, which are consistent with the requirements for developing α-glucosidase inhibitors. Consequently, this study selected chlorogenic acid as a positive control to examine the inhibitory effects of polyphenol extracts on α-glucosidase. As illustrated in Figure 4, the inhibition rates of chlorogenic acid and honeysuckle leaf polyphenol extracts on α-glucosidase were significantly and positively correlated with their concentrations, indicating that both compounds exhibited pronounced dose-dependent inhibitory effects. The increase in polyphenol content diminished the binding of α-glucosidase to the substrate PNPG, thereby preventing the formation of the colored product, p-nitrophenol, and consequently exerting an inhibitory effect. Through fitting calculations, the IC_50_ values for chlorogenic acid and polyphenol extracts were determined to be 0.55 ± 0.02 mg/mL and 0.14 ± 0.01 mg/mL. Puspitasari et al. (35) determined the IC_50_ of acarbose, a commonly used anti-diabetic drug, to be 2.4 ± 0.2 mg/mL. Therefore, the polyphenolic extract from the leaves of honeysuckle presents a promising potential as an α-glucosidase inhibitor, with valuable applications in functional foods and pharmaceuticals.

### 3.5 Combing behavioral analysis

**Figure 5:**
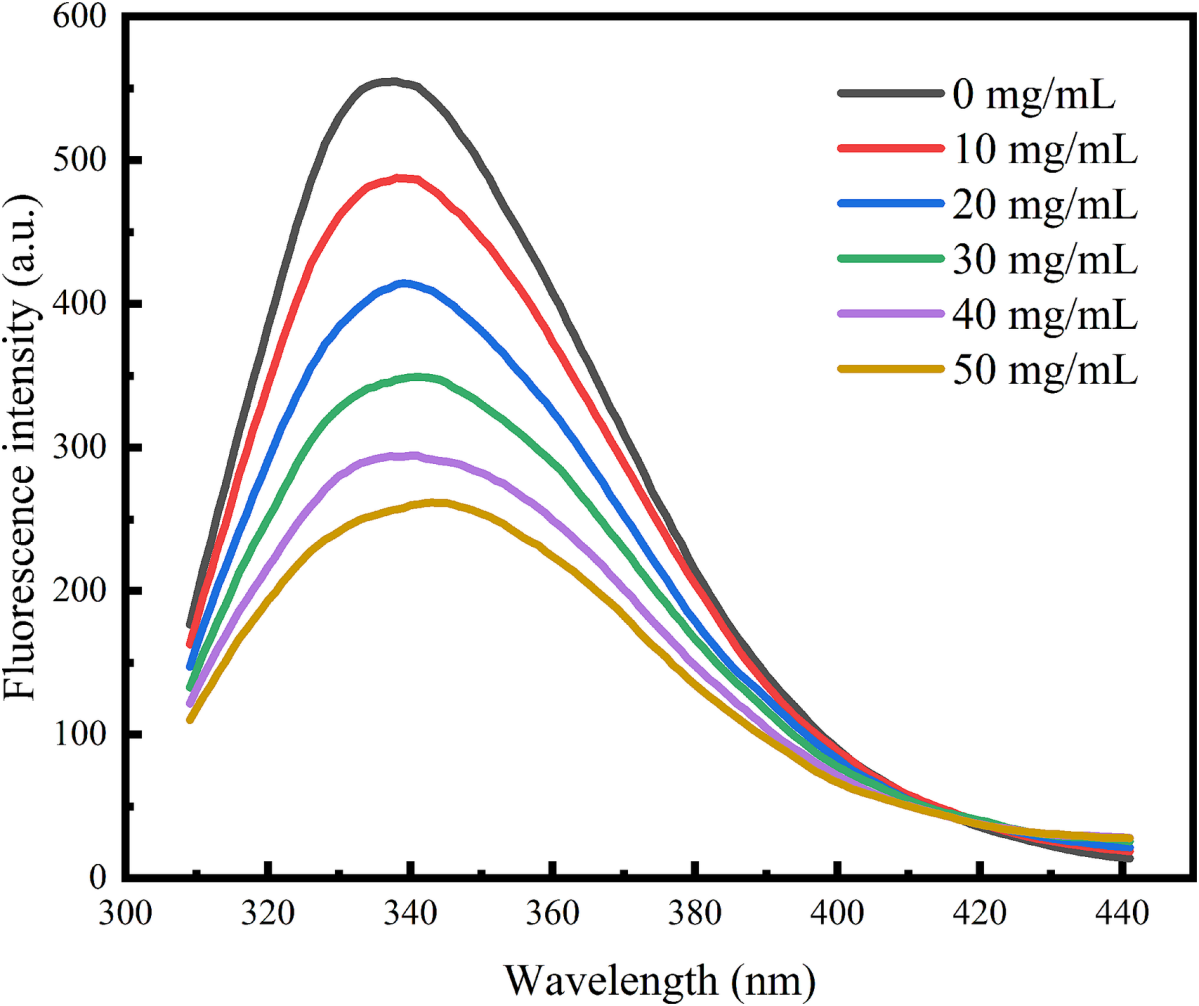
Effect of polyphenol extract on fluorescence spectra of α-glucosidase.

Fluorescence spectroscopy serves as an effective technique for investigating the interactions between small drug molecules and enzymes. These interactions can modify the microenvironment of amino acid residues, resulting in changes in fluorescence intensity. As illustrated in Figure 5, when the excitation wavelength was set to 280 nm, the fluorescence spectra of the tyrosine and tryptophan residues of the enzyme were recorded. Following the addition of polyphenol extracts, a consistent decrease in the fluorescence intensity of the enzyme was observed. Notably, α-glucosidase displayed a characteristic fluorescence absorption peak at 338 nm; as the concentration of polyphenol extract increased, the fluorescence spectrum exhibited a red shift. This observation indicates that an interaction occurred between the enzyme and the polyphenol extracts, leading to alterations in the microenvironment of the amino acid residues. Liu et al. (36) demonstrated in their experiments that this alteration may hinder the binding of α-glucosidase to its substrate, thereby reducing enzyme activity and effectively lowering postprandial hyperglycemia.

### 3.6 Synchronous fluorescence spectroscopy analysis

**Figure 6:**
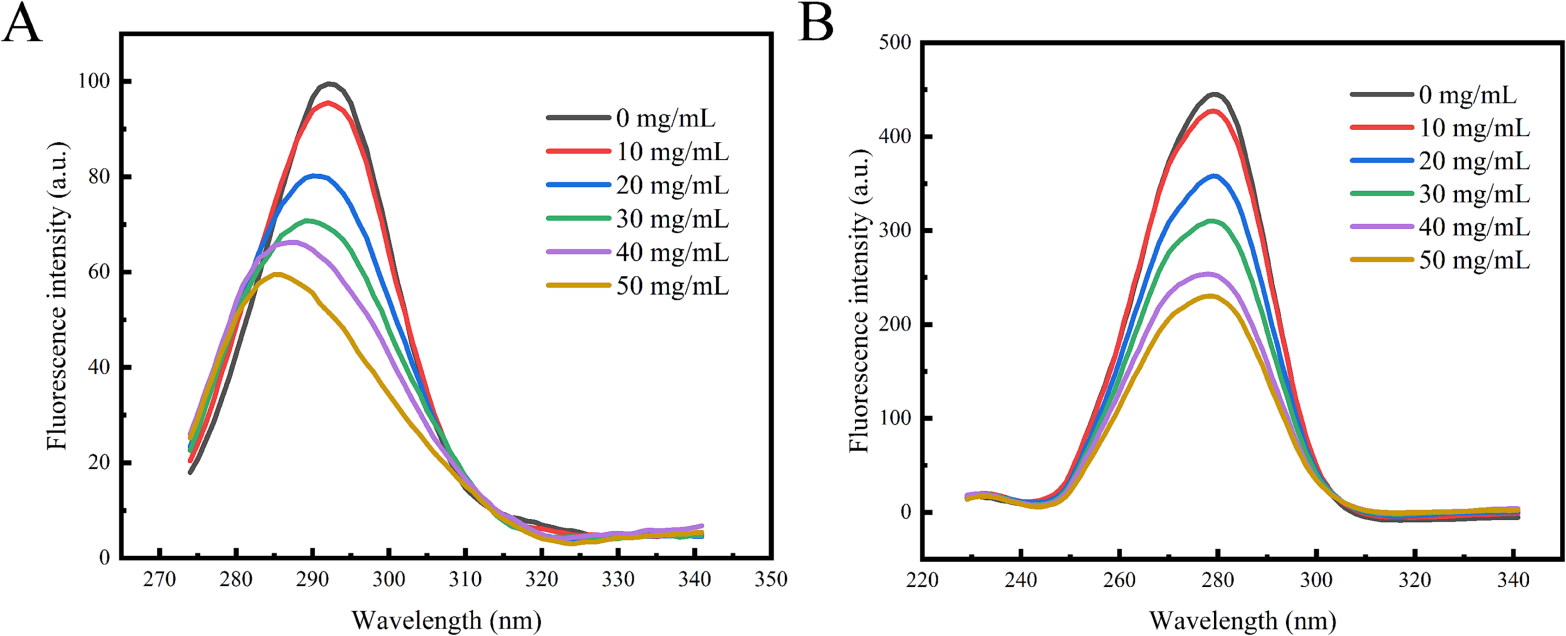
Effect of polyphenol extract on synchronous fluorescence spectra of α-glucosidase. **(A)** The synchronous spectrum at Δ*λ* = 15 nm. **(B)** The synchronous spectrum at Δ*λ* = 60 nm.

By setting Δ*λ* to 15 nm and 60 nm, the characteristic fluorescence peaks of tyrosine and tryptophan residues were identified. As illustrated in Figure 6, the synchronous fluorescence spectra of these two amino acid residues exhibit a consistent decrease with increasing concentrations of polyphenol extract. The fluorescence intensity of tryptophan was greater than that of tyrosine, and its quenching rate was also faster, which aligns with findings from previous studies (37). Notably, the characteristic peak of tyrosine underwent significant alterations, suggesting that the polyphenol extract interacts with α-glucosidase, leading to changes in the microenvironment of the amino acid residues. Consequently, it can be inferred that the polyphenolic extract from honeysuckle may bind to the active site or adjacent regions of α-glucosidase, inducing conformational changes in the enzyme. This binding likely prevents the substrate from entering the active site, thereby exerting an inhibitory effect on α-glucosidase and resulting in a decrease in its activity.

### 3.7 3D fluorescence spectroscopy analysis

**Figure 7:**
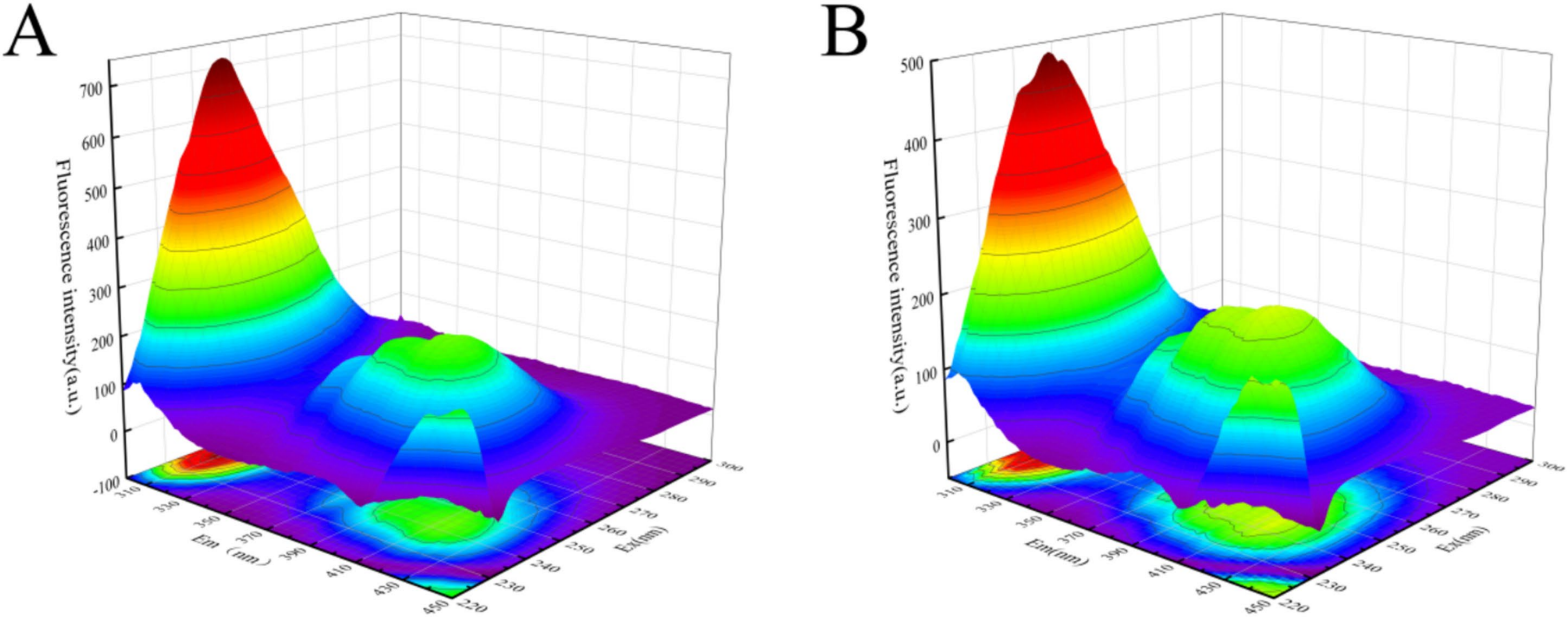
Effect of polyphenol extract on three-dimensional fluorescence spectra of α-glucosidase. **(A)** Three-dimensional fluorescence spectra of α-glucosidase. **(B)** Three-dimensional fluorescence spectra of α-glucosidase after addition of polyphenol extract.

Three-dimensional fluorescence spectroscopy was employed to further investigate the conformational changes of α-glucosidase induced by polyphenol extracts. As illustrated in Figure 7A, the 3D fluorescence spectrum of α-glucosidase displayed two characteristic peaks. Peak 1 (*λ*_EX_/*λ*_EM_ = 225 nm/338 nm) corresponded to the spectral behavior resulting from *n* → π* transitions within the polypeptide backbone structure (38), whereas Peak 2 (*λ*_EX_/*λ*_EM_ = 280 nm/336 nm) represented the fluorescence spectra of tyrosine and tryptophan residues (39). Following the complexation with polyphenol extracts, the fluorescence intensities of both Peak 1 and Peak 2 were significantly diminished, as depicted in Figure 7B. The emission wavelengths of Peak 1 and Peak 2 both exhibited a slight redshift, with Peak 1 shifting from 338 nm to 400 nm and Peak 2 from 336 nm to 338 nm. These results further corroborated the interaction between the polyphenol extracts and α-glucosidase, indicating that the polyphenol extracts bind to regions near the tryptophan and tyrosine residues, thereby influencing the conformational structure of the enzyme’s polypeptide chain.

## 4 Conclusion

This study utilized ultrasound-assisted extraction to obtain polyphenols from honeysuckle leaves. The optimal extraction conditions were identified as an ethanol concentration of 64%, a liquid–solid ratio of 45 mL/g, an ultrasonic time of 6 min, and an ultrasonic power of 700 W. The actual yield of polyphenols was 9.16 ± 0.19%, which closely approximated the predicted value of 9.18%. The total phenolic content of the extracts was measured at 20.6 ± 0.67%, with chlorogenic acid and luteoloside contents recorded at 5.65 ± 0.40% and 2.51 ± 0.14%, respectively. The polyphenol extract demonstrated significant α-glucosidase inhibitory activity (IC_50_, 0.14 ± 0.01 mg/mL), surpassing that of chlorogenic acid (IC_50_, 0.55 ± 0.02 mg/mL). Fluorescence quenching experiments indicated that the polyphenol extracts interacted with α-glucosidase, modifying its microenvironment and reducing its substrate binding, thereby effectively mitigating postprandial hyperglycemia. This experiment laid the foundation for the study of honeysuckle leaf polyphenols, and further in-depth research on the biological activity and potential synergistic effect of its polyphenol compounds can be carried out in the future.

## Data Availability

The original contributions presented in the study are included in the article/supplementary material, further inquiries can be directed to the corresponding author.
